# Examining recent effects of caffeine on default mode network and dorsal attention network anticorrelation in youth

**DOI:** 10.1371/journal.pone.0327385

**Published:** 2025-07-02

**Authors:** Orrin D. Ware, Sarah E. Chang, Wesley K. Thompson, Alexandra S. Potter, Hugh Garavan, Micah E. Johnson, Lucina Q. Uddin

**Affiliations:** 1 School of Social Work, University of North Carolina at Chapel HillChapel Hill, North Carolina, United States of America; 2 Department of Psychiatry and Biobehavioral Sciences, University of California Los Angeles, California, United States of America; 3 Center for Population Neuroscience and Genetics, Laureate Institute for Brain Research, Tulsa, Oklahoma, United States of America; 4 Department of Psychiatry, University of Vermont, Burlington, Vermont, United States of America; 5 Department of Family Medicine, University of California Los Angeles, Los Angeles, California, United States of America; 6 Department of Psychology, University of California Los Angeles, Los Angeles, California, United States of America; University of Turin: Universita degli Studi di Torino, ITALY

## Abstract

**Objectives:**

In adolescence, caffeinated beverage consumption is negatively associated with cognitive functioning. The default mode network and dorsal attention network are anticorrelated brain systems that are essentially implicated in attention. Despite the importance of the anticorrelation of default mode network – dorsal attention network on cognitive functioning, no studies have examined the association between this anticorrelation and recent caffeine consumption among youths. This study analyzed baseline data from the Adolescent Brain Cognitive Development℠ Study, the largest longitudinal study examining brain development and adolescent health in the United States, to explore the associations between caffeinated beverage consumption and the strength of anticorrelation between the default mode network – dorsal attention network.

**Methods:**

A total of N = 4,673 early adolescents (average age 9.9 years, standard deviation = 0.6) had self-report data for two caffeine variables: [a] last 24-hour caffeinated beverage consumption (Yes/No) and [b] weekly caffeinated beverage consumption (continuous). A mixed-effects model was fitted with default mode network – dorsal attention network anticorrelation strength as the outcome.

**Results:**

Most of the baseline ABCD sample did not consume a caffeinated beverage in the last 24 hours (n = 3,910; 83.7%). Controlling for covariates (age, attention problems, BMI, family, head motion, MRI scanner, and sex), neither the caffeinated beverage variables nor their interaction were statistically significant.

**Conclusions:**

Our study findings identified that approximately 16% of our sample consumed caffeine in the last 24 hours prior to the magnetic resonance imaging scan. We did not find caffeine to impact the default mode network – dorsal attention network anticorrelation strength in this sample. This study may guide the interpretation of functional magnetic resonance imaging results among adolescents who consume caffeinated beverages.

## Introduction

Caffeine, or 1,3,7-Trimethylxanthine [1], is one of the most widely consumed psychoactive substances both globally and in the United States [2–4]. Although caffeine can be consumed in a variety of ways, caffeinated beverages (e.g., coffee, energy drinks, soda, tea) are among the most common [1,5]. As a psychostimulant, caffeine acts on the central nervous system of individuals who consume the substance [6,7]. Overall, caffeine is a highly popular substance that has a direct impact on cognitive functioning [8].

Evidence suggests that many youths consume caffeinated beverages [9–11]. Dietary data from a nationally representative sample suggests that approximately 70% of adolescents ingest caffeine on a given day [11]. Evidence suggests that caffeine may affect the cognitive functioning of youths [12]. Data from the Adolescent Brain Cognitive Development℠ Study (ABCD Study^®^), the largest longitudinal study examining brain development and adolescent health in the United States [13], identified caffeine intake as negatively associated with cognitive flexibility, processing speed, and episodic memory in 9–10 year olds [12]. Considering caffeine’s prevalence and potential impact on these cognitive domains in youth, it is imperative to examine the extent to which neuroimaging variables, such as metrics derived from resting-state functional magnetic resonance imaging (fMRI) data, are potentially influenced by caffeine.

Resting-state fMRI provides the opportunity to examine the brain’s intrinsic functional organization [14,15]. This functional architecture consists of large-scale networks that exhibit spontaneous, correlated activity, including the default mode network (DMN) and dorsal attention networks (DAN). DMN is thought to support internally-directed processes (e.g., reflection, mind wandering), whereas the DAN supports top-down attention. A cogent body of literature suggests that the default mode network (DMN) exhibits negative correlation (anticorrelation) with task-positive networks such as the dorsal attention network (DAN) during goal-directed tasks and at rest [15–20]. A greater anticorrelation value between the DMN and DAN is indicative of greater segregation between the two networks, wherein more negative values indicate greater anticorrelation (stronger negative correlation). Individual differences in the strength of anticorrelation between the DMN-DAN were first shown to be significantly related to response time variability (indicating better cognitive performance/attention in adults, a finding that was later replicated in early adolescence [21–23]. In these studies, greater DMN-DAN anticorrelation was associated with more consistent behavioral performance. There is now a large body of work mostly conducted in adults linking DMN anticorrelations and cognitive function in the domains of attention, executive function, and cognitive control (see Tripathi for review) [24].

In adults, studies indicate that caffeine consumption can produce widespread changes in functional connectivity [25], cerebral blood flow [26], and blood oxygenation level-dependent (BOLD) signal [27]. The earliest study to examine the influence of caffeine on resting state functional connectivity reported that caffeine consumption reduced functional connectivity in the motor cortex [28]. Despite the importance of the anticorrelation of DMN-DAN on cognitive functioning, especially attention [21,22], we were unable to identify studies that examined the association between caffeine consumption and the anticorrelation of DMN-DAN among youths. Considering caffeine can improve cognitive performance in adults [8,29], yet decrease cognitive functioning in youths [12], it is important to examine whether the same functional brain networks are impacted in early adolescents when they consume caffeine. Youths may be more vulnerable to the negative drug-effects of caffeine because of their developing nervous system and potential lower tolerance [30,31]. Further, an adult may decide to ingest caffeine specifically to remain awake and improve attention whereas, youths may decide to ingest caffeine found in a soda or tea without the explicit purpose of remaining awake [32,33]. This consumption may unintendedly impact the sleep patterns of these youths, increasing the potential negative drug effects [32]. The current study is the first to examine the associations between both self-reported past 24 hour caffeine consumption and weekly caffeinated beverage consumption and the anticorrelation of DMN-DAN among a sample of early adolescents. The motivations for these two questions are to provide insight into whether regular caffeine consumption among adolescents is a potential confounder for the anticorrelation of DMN-DAN. Evidence suggests that fMRI can detect the effect of drugs on an individual’s brain for days to weeks of drug administration [25,34–37]. While researchers have identified caffeine’s impact on youth cognitive functioning using cognitive tasks in the ABCD Study [12], the current study focuses on fMRI to examine caffeine’s potential effect on the brain’s functional organization. Based on prior work in adults, we hypothesized that caffeine intake would be associated with greater DMN-DAN anticorrelation [38].

## Materials and methods

### Data and sample selection

This study analyzed data from the ABCD Study^®^ data release 5.1. The ABCD Study^®^ captures data across 21 research sites among 11,878 adolescents aged 9–10 at the baseline study visit [13]. Neuroimaging, weekly caffeinated beverage consumption, and last 24-hour caffeinated beverage consumption data were examined for the current study. Sample selection criteria were: [a] on-site interview, [b] variables captured on the same date, [c] no missing data, and [d] body mass index (BMI) score of ≤ 100 (to exclude severe pathological conditions). The sample meeting the criteria includes N = 4,673 youths, with data representing the baseline timepoint captured from 2017 to 2018. Each study site collected informed consent from parents and guardians and assent from children, approved by each site’s Institutional Review Board (IRB), with centralized IRB approval at the University of California, San Diego. This current study was considered non-human subjects research by the University of North Carolina at Chapel Hill IRB. This current study, which is a secondary analysis of de-identified data, did not require interaction with study participants or obtaining consent.

### Measures

Primary study variables included: [a] last 24-hour caffeinated beverage consumption, [b] weekly caffeinated beverage consumption, and [c] average anticorrelation between DMN-DAN. This study also included covariates: [a] age, [b] attention problems, [c] BMI, [d] family, [e] head motion, [f] MRI scanner, and [g] sex. A supplemental analysis included a past 12-month combined family income variable.

### Last 24 hour caffeinated beverage consumption

Adolescents were asked if they ingested a caffeinated beverage in the last 24 hours with binary responses: Yes/No.

### Weekly caffeinated beverage consumption

Adolescents were asked if they had heard of caffeine. If they heard of caffeine, they were asked if they consumed caffeinated beverages. Consuming caffeinated beverages prompts questions about how many of the following caffeinated beverages they consumed per week in the last 6 months: [a] coffee, [b] espresso/espresso containing drinks, [c] tea, [d] soda, and [e] energy drinks. These five beverages were summed.

### Average anticorrelation between default mode network and dorsal attention network

**Fig 1:**
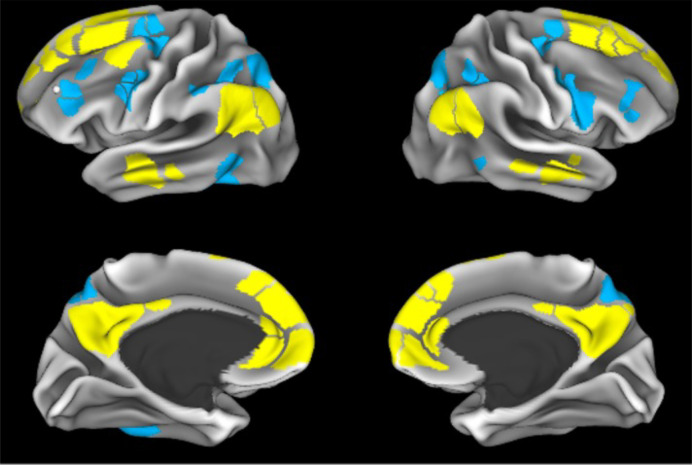
Visualization of the default mode network (yellow) and dorsal attention network (blue) from Gordon et al. 2016.

Fig 1 shows the DMN and DAN from the Gordon parcellation, as provided in the tabulated data provided by the ABCD Study^®^. Details of data acquisition and centralized processing for the ABCD Study^®^ have been previously detailed [39,40], including T1-weighted, T2-weighted, and resting state imaging that was used in computing the DMN-DAN anticorrelation. Pairwise correlations between each region of interest (ROI) within the DMN and each ROI in the DAN, as identified in the Gordon parcellation, were utilized [17]. These between-network correlations were averaged and Fisher-Z transformed to generate a summary metric of overall anticorrelation between DMN-DAN, wherein more negative values indicate greater anticorrelation (stronger negative correlation).

### Covariates

Age was expressed in months. Attention problems were indexed by t-scores of the Child Behavior Checklist: Attention problems subscale, a parent-report questionnaire [41]. Height and weight data were used to create categorical BMI: [a] Underweight, [b] Normal weight, [c] Overweight, and [d] Obese [42]. Family described if individuals were in the same family using a unique code, with 4,061 different families in the current study. Head motion was indexed by average framewise displacement in millimeters during the resting state fMRI scans. MRI scanner described the specific imaging device serial number used to conduct the neuroimaging with 27 different scanners collecting data used for this study. Parents of the respondents were asked the respondent’s sex at birth, with [a] female, [b] intersex, and [c] male as responses.

### Analysis

R version 4.3.3 (R Core Team) [43] and, IBM Statistical Package for the Social Sciences Version 29 software was used for this study [44]. Using the “lme4” package [45], a mixed-effects model was fitted to examine relationships between caffeine consumption and DMN-DAN anticorrelation. Bivariable analyses using t-tests and Chi-Square were conducted. T-test assumptions were checked using Q-Q plots, Shapiro-Wilk tests, Kolomogorov-Smirnov tests, and groups were checked for equal variance. Although the continuous variables were not normally distributed based on the assumption checking, the sample size was sufficient to proceed with t-tests. However, nonparametric tests were also conducted to examine whether medians of the continuous variables were the same across the binary caffeine consumption groups. A mixed effects model, which is a type of model that allows for the inclusion of random effects, was selected to include family as a random intercept. The model included the fixed-effect covariates, and independent variables of interest. A second mixed-effects model that only included the covariates was conducted, and a likelihood ratio test was used to test for the effects of the caffeine variables. These two separate models permitted us to examine the potential impact of the caffeine variables when added alongside the covariates. Statistical significance was determined as p < .05, and no corrections for multiple comparisons were conducted. No corrections for multiple comparisons were conducted because this study focused primarily on the caffeine variables as described in our hypothesis. To test our hypothesis, that caffeine intake would be associated with greater DMN-DAN anticorrelation, we included the covariate model and the model that tested the caffeine consumption variables alongside covariates. The “plot” function was used to examine and ultimately identify homogeneity of variance in both models. The “hist” function was used to examine and ultimately identify the random distribution of errors in both models. Age was added as a covariate because data suggests that older youths typically ingest more caffeine than younger youths [11,46]. BMI and sex were included in the model because a national nutritional study among youth has shown associations between caffeine consumption, gender, and BMI [47]. The attention problems variable was added as a covariate to examine if there was a relationship between the dependent variable attributable to caffeine consumption or attention problems. Supplemental bivariable analyses explored parent-reported past 12-month combined family income with the caffeine variables. These findings may be found in Supplemental Document.

## Results

Table 1 shows sample characteristics and bivariable analyses. The sample was an average of 118.5 months old (standard deviation [SD] = 8.0) or 9.9 years old (SD = 0.6) and consisted of a slight majority of males (n = 2,433; 52.1%). Most of the sample did not consume a caffeinated beverage in the last 24 hours (n = 3,910; 83.7%). Slightly more than a quarter (n = 1,342; 28.7%) reported 0 for their weekly caffeinated beverage consumption. Of the n = 3,331 (71.3%) that reported > 0 for their weekly caffeinated beverage consumption, an average of 3.4 (SD = 9.4) caffeinated beverages were reported. Mood’s independent-samples median nonparametric test identified age as not being statistically different between the two groups (p = .406). However, the BMI numerical (χ^2^ = 34.851; p < .001), weekly caffeinated beverage consumption (χ^2^ = 418.147; p < .001), attention (χ^2^ = 12.619; p < .001), and average anticorrelation between DMN-DAN (χ^2^ = 11.361; p < .001) variables were significant.

**Table 1:** Characteristics of the study sample, N = 4,673.

	Entire Study Sample, N = 4,673	Had a Caffeinated Beverage in the last 24 hours, n = 763	Did not have a Caffeinated Beverage in the Last 24 Hours, n = 3,910	Between Group Differences
	**Mean (SD)**	**Mean (SD)**	**Mean (SD)**	**t (p)**
Age, in months	118.5 (7.5)	118.8 (7.7)	118.5 (7.5)	−1.1 (.283)^1^
Body Mass Index Numerical Value	18.8 (4.4)	19.7 (4.8)	18.7 (4.3)	−6.3 (<.001)^2^
Weekly Caffeinated Beverage Consumption	2.4 (8.0)	5.3 (11.9)	1.8 (6.9)	−11.2 (<.001)^3^
Attention Problems	53.2 (5.7)	54.1 (6.6)	53.1 (5.4)	−4.5 (<.001)^4^
Average Anticorrelation between Default Mode Network and Dorsal Attention Network	−8.145041e-16 (1.0)	0.047024 (1.0)	−0.009176 (1.0)	−1.4 (.156)^5^
	**n (%)**	**n (%)**	**n (%)**	**χ**^2^ **(p)**
Caffeinated Beverage in the last 24 hours				
Yes	763 (16.3)	763 (100.0)	0 (0.0)	
No	3,910 (83.7)	0 (0.0)	3,910 (100.0)	
Sex				6.7 (.009)^6^
Female	2,240 (47.9)	333 (43.6)	1,907 (48.8)	
Male	2,433 (52.1)	430 (56.4)	2,003 (51.2)	
Body Mass Index				44.9 (<.001)^7^
Underweight	2,787 (59.6)	375 (49.1)	2,412 (61.7)	
Normal weight	1,444 (30.9)	286 (37.5)	1,158 (29.6)	
Overweight	331 (7.1)	74 (9.7)	257 (6.6)	
Obese	111 (2.4)	28 (3.7)	83 (2.1)	

^1^Cohen’s d: Estimate = -.043; Confidence Interval = -.120,.035

^2^Cohen’s d: Estimate = -.251; Confidence Interval = -.328, -.173

^3^Cohen’s d: Estimate = -.444; Confidence Interval = -.522, -.366

^4^Cohen’s d: Estimate = -.177; Confidence Interval = -.254, -.099

^5^Cohen’s d: Estimate = -.056; Confidence Interval = -.133,.023

^6^Cramer V =.038; Sig =.009

^7^Cramer V =.098; Sig = <.001

Results from the mixed-effects model can be found in Table 2. Controlling for covariates, the caffeinated beverage variables were not significant [a] last 24 hours (Estimate = −0.02; 95% Confidence Interval [CI] = −0.10, 0.06), [b] weekly (Estimate = 0.00; 95% CI = −0.00, 0.01) [c] interaction between caffeine variables (Estimate = −0.00; 95% CI = −0.01, 0.00). Results from the mixed-effects model that only included covariates, which is found in Table 3, and the likelihood ratio test, which is found in Table 4, provide further support that including the caffeine variables does not impact DMN-DAN anticorrelation. During the peer review process, it was suggested that BMI be added to the mixed effects model as a continuous variable instead of a categorical variable. The two caffeine consumption variables and their interaction were non-significant in this supplemental analysis. These findings may also be found in the Supplemental Document.

**Table 2:** Mixed-effects model examining the association between caffeinated beverage consumption, covariates and the correlation between default mode network and dorsal attention network, N = 4,673.

Variable	Estimate	Standard Error	Confidence Interval	p
Intercept	0.28	0.26	−0.24–0.79	0.289
Caffeinated beverage in last 24 hours	−0.02	0.04	−0.10–0.06	0.595
Weekly caffeinated beverage consumption	0.00	0.00	−0.00–0.01	0.320
Caffeinated beverage in last 24 hours x weekly caffeinated beverage consumption	−0.00	0.00	−0.01–0.00	0.331
Age	−0.01	0.00	−0.01 – −0.00	**<0.001**
Sex	−0.18	0.03	−0.23 – −0.12	**<0.001**
Attention Problems	0.01	0.00	0.01–0.02	**<0.001**
BMI: underweight	−0.08	0.03	−0.14 – −0.02	**0.010**
BMI: overweight	0.05	0.06	−0.06–0.16	0.327
BMI: obese	−0.11	0.09	−0.28–0.07	0.241
Head motion	0.58	0.04	0.51–0.66	**<0.001**
Scanner #1	−0.53	0.09	−0.70 – −0.35	**<0.001**
Scanner #2	−0.41	0.09	−0.58 – −0.24	**<0.001**
Scanner #3	−0.26	0.09	−0.45 – −0.08	**0.005**
Scanner #4	0.22	0.26	−0.30–0.74	0.404
Scanner #5	−0.57	0.08	−0.72 – −0.42	**<0.001**
Scanner #6	−0.20	0.09	−0.37 – −0.02	**0.025**
Scanner #7	−0.50	0.09	−0.67 – −0.33	**<0.001**
Scanner #8	0.56	0.09	0.37–0.74	**<0.001**
Scanner #9	−0.23	0.10	−0.42 – −0.04	**0.018**
Scanner #10	0.30	0.11	0.09–0.51	**0.006**
Scanner #11	0.02	0.09	−0.15–0.19	0.844
Scanner #12	0.11	0.10	−0.09–0.31	0.288
Scanner #13	0.03	0.10	−0.16–0.22	0.767
Scanner #14	0.02	0.09	−0.15–0.19	0.809
Scanner #15	−0.18	0.14	−0.46–0.10	0.207
Scanner #16	−0.30	0.09	−0.48 – −0.13	**0.001**
Scanner #17	0.26	0.11	0.04–0.47	**0.019**
Scanner #18	−0.50	0.25	−1.00–0.00	0.051
Scanner #19	−0.00	0.09	−0.19–0.18	0.965
Scanner #20	−0.82	0.92	−2.62–0.98	0.369
Scanner #21	−0.25	0.08	−0.40 – −0.09	**0.002**
Scanner #22	0.13	0.10	−0.07–0.34	0.188
Scanner #23	0.19	0.10	−0.01–0.39	0.061
Scanner #24	0.48	0.11	0.25–0.70	**<0.001**
Scanner #25	−0.05	0.08	−0.20–0.10	0.521
Scanner #26	0.23	0.10	0.04–0.42	**0.017**

**Table 3:** Mixed-effects model examining the association between covariates and the correlation between default mode network and dorsal attention network: caffeine variables excluded, N = 4,673.

Variable	Estimate	Standard Error	Confidence Interval	p
Intercept	0.27	0.26	−0.24–0.79	0.297
Age	−0.01	0.00	−0.01 – −0.00	**<0.001**
Sex	−0.17	0.03	−0.23 – −0.12	**<0.001**
Attention Problems	0.01	0.00	0.01–0.02	**<0.001**
BMI: underweight	−0.08	0.03	−0.14 – −0.02	**0.011**
BMI: overweight	0.05	0.06	−0.06–0.16	0.330
BMI: obese	−0.11	0.09	−0.28–0.07	0.241
Head motion	0.58	0.04	0.51–0.66	**<0.001**
Scanner #1	−0.52	0.09	−0.70 – −0.35	**<0.001**
Scanner #2	−0.40	0.09	−0.57 – −0.23	**<0.001**
Scanner #3	−0.26	0.09	−0.45 – −0.08	**0.005**
Scanner #4	0.22	0.26	−0.29–0.74	0.399
Scanner #5	−0.57	0.08	−0.72 – −0.42	**<0.001**
Scanner #6	−0.20	0.09	−0.37 – −0.02	**0.028**
Scanner #7	−0.49	0.08	−0.66 – −0.33	**<0.001**
Scanner #8	0.57	0.09	0.38–0.75	**<0.001**
Scanner #9	−0.23	0.10	−0.42 – −0.04	**0.018**
Scanner #10	0.30	0.11	0.09–0.51	**0.005**
Scanner #11	0.02	0.09	−0.15–0.19	0.787
Scanner #12	0.12	0.10	−0.08–0.32	0.253
Scanner #13	0.03	0.10	−0.16–0.22	0.749
Scanner #14	0.02	0.09	−0.15–0.19	0.814
Scanner #15	−0.17	0.14	−0.45–0.11	0.229
Scanner #16	−0.30	0.09	−0.47 – −0.13	**0.001**
Scanner #17	0.26	0.11	0.04–0.48	**0.018**
Scanner #18	−0.50	0.25	−0.99–0.00	0.052
Scanner #19	−0.00	0.09	−0.18–0.18	0.992
Scanner #20	−0.82	0.92	−2.62–0.98	0.373
Scanner #21	−0.24	0.08	−0.40 – −0.08	**0.003**
Scanner #22	0.14	0.10	−0.06–0.34	0.172
Scanner #23	0.19	0.10	−0.01–0.39	0.058
Scanner #24	0.48	0.11	0.26–0.71	**<0.001**
Scanner #25	−0.05	0.08	−0.20–0.10	0.532
Scanner #26	0.23	0.10	0.05–0.42	**0.015**

**Table 4:** Likelihood ratio test for mixed effects models.

	npar	AIC	BIC	LogLik	deviance	Chisq	Df	p
Covariate Only Model	36	12438	12670	−6183.1	12366			
Caffeine with Covariate Model	39	12442	12694	−6182.1	12364	1.9852	3	0.5755

## Discussion

DMN-DAN anticorrelation is an index of brain network segregation that has been implicated in attention and shows developmental maturation patterns linked to better cognitive performance [21]. Caffeine impacts measures of brain functional connectivity [28]. While data are lacking regarding how caffeine affects the brain in adolescence, we know that caffeine impacts cognitive functioning [12]. The current study is the first to examine the association between past 24 hour and weekly caffeine intake and the anticorrelation of DMN-DAN among early adolescents.

When examined alongside covariates, this study did not find any association between self-reported past 24 hour and weekly caffeine consumption and the anticorrelation of DMN-DAN. These null findings have real-world implications regarding fMRI scans and the anticorrelation of DMN-DAN. Our study findings identified that approximately 16% of the sample consumed a caffeinated beverage in the last 24 hours prior to the MRI scan. Since some adolescents may consume caffeine before an MRI scan and results from this study did not find caffeine to impact the DMN-DAN anticorrelation, this study may guide the interpretation of MRI results among adolescents who consume caffeinated beverages.

Covariates that were associated with the anticorrelation of DMN-DAN in the full model included age, female sex, attention problems, being underweight, head motion, and specific scanners. Although this study only includes nine and 10-year-olds, the age findings confirm that older age among youths is associated with increased DMN-DAN anticorrelation [23,48]. Similarly, this study identifies being female sex as having greater DMN-DAN anticorrelation compared to males, which confirms findings from other studies [23,49]. Since greater DMN-DAN anticorrelation is associated with better cognitive performance/attention [21–23], our findings suggest that attention problems and head motion were associated with diminished DMN-DAN anticorrelation seems appropriate. Being underweight was associated with diminished DMN-DAN anticorrelation compared to being of normal weight. More studies are needed to further examine the relationship between BMI and DMN-DAN among adolescents.

It is important to note that the tabulated ABCD data were analyzed using a pipeline that includes global signal regression (GSR), a preprocessing step whereby the average value of all whole-brain signals is regressed from the data prior to analyses. GSR is a powerful tool for removing MRI artifacts due to participant motion, respiration, and physiological noise [50], but also can mathematically introduce negative correlations into the data [51]. The DMN-DAN anticorrelation has been observed in studies both with and without GSR [52], but an important future direction would be to examine whether this analytic decision influences the current results. Another important consideration is that caffeine consumption leads to widespread decreases in functional connectivity and global signal amplitude, leading to significant enhancement of detection of DMN-DAN anticorrelations [38]. It is thus somewhat surprising that we observe no effects of caffeine on this anticorrelation in the current sample, however this could be due to the low dosages reported by children at age 9–10 in the ABCD Study.

### Limitations

While this study provides details regarding the association between caffeine and the DMN-DAN anticorrelation in youth, findings should be interpreted alongside study limitations. These include: self-report data increasing the risk of response bias, other potential covariates that could influence the model, meeting the sample inclusion criteria (e.g., no incomplete data for any variables included in this analysis), and retrospectively capturing the number of caffeinated beverages instead of prospectively capturing caffeine intake in milligrams. There is variability in the milligrams of caffeine within specific beverage types such as energy drinks, sodas, and teas. Therefore, future studies are needed with data containing these beverages’ caffeine milligram content. Further, no data identifies the age of onset for a participant’s regular caffeine consumption. There is a temporal limitation in that while participants may report consuming caffeine in the past 24 hours, it was unclear whether each participant responded to this question before or after the scan. If responses preceded the scan there is possibility that participants may have consumed caffeine between the questionnaire administration and the scan itself. Finally, DMN anticorrelations have been associated with additional networks outside of the DAN (see Tripathi for review) [24]. Thus, another limitation is that other network relationships (e.g., DMN-frontoparietal or DMN-salience) were not examined here, which may be explored in future studies.

## Supporting information

Supplemental Table 1 Mixed-Effects Model Examining the Association Between Caffeinated Beverage Consumption, Covariates and the Correlation between Default Mode Network and Dorsal Attention Network, BMI as a continuous variable, N = 4,673. (DOCX)

Supplemental Table 2 Income and Caffeinated Beverage in the last 24 hours, N = 4,295. (DOCX)

Supplemental Table 3 Income and Weekly Caffeinated Beverage Consumption, N = 4,295. (DOCX)
