# Supplementary material for: Examining recent effects of caffeine on default mode network and dorsal attention network anticorrelation in youth
Source: PLoS One. 2025 Jul 2;20(7):e0327385. doi: 10.1371/journal.pone.0327385 (PMC12221008; doi:10.1371/journal.pone.0327385)
Supplement: Supplemental Table 1 — (DOCX) [file pone.0327385.s001.docx]

**Supplemental Document: Supplemental Table 1**

| **Supplemental Table 1. Mixed-Effects Model Examining the Association Between Caffeinated Beverage Consumption, Covariates and the Correlation between Default Mode Network and Dorsal Attention Network, BMI as a continuous variable, N = 4,673** |  |  |  |  |
| --- | --- | --- | --- | --- |
| **Variable** | **Estimate** | **Standard**  **Error** | **Confidence Interval** | **p** |
| Intercept | 0.07 | 0.26 | -0.45 – 0.58 | 0.797 |
| Caffeinated beverage in last 24 hours | -0.02 | 0.04 | -0.10 – 0.06 | 0.651 |
| Weekly caffeinated beverage consumption | 0.00 | 0.00 | -0.00 – 0.01 | 0.353 |
| Caffeinated beverage in last 24 hours x weekly caffeinated beverage consumption | -0.00 | 0.00 | -0.01 – 0.00 | 0.312 |
| Age | -0.01 | 0.00 | -0.01 – -0.00 | **<0.001** |
| Sex | -0.17 | 0.03 | -0.23 – -0.12 | **<0.001** |
| Attention Problems | 0.01 | 0.00 | 0.01 – 0.02 | **<0.001** |
| BMI: continuous | 0.01 | 0.00 | 0.00 – 0.01 | **0.018** |
| Head motion | 0.59 | 0.04 | 0.51 – 0.66 | **<0.001** |
| Scanner #1 | -0.53 | 0.09 | -0.70 – -0.35 | **<0.001** |
| Scanner #2 | -0.40 | 0.09 | -0.57 – -0.23 | **<0.001** |
| Scanner #3 | -0.26 | 0.09 | -0.44 – -0.07 | **0.006** |
| Scanner #4 | 0.23 | 0.26 | -0.29 – 0.74 | 0.392 |
| Scanner #5 | -0.57 | 0.08 | -0.72 – -0.42 | **<0.001** |
| Scanner #6 | -0.19 | 0.09 | -0.37 – -0.02 | **0.032** |
| Scanner #7 | -0.49 | 0.09 | -0.66 – -0.32 | **<0.001** |
| Scanner #8 | 0.56 | 0.09 | 0.38 – 0.75 | **<0.001** |
| Scanner #9 | -0.22 | 0.10 | -0.41 – -0.03 | **0.023** |
| Scanner #10 | 0.30 | 0.11 | 0.10 – 0.51 | **0.004** |
| Scanner #11 | 0.02 | 0.09 | -0.15 – 0.20 | 0.781 |
| Scanner #12 | 0.11 | 0.10 | -0.09 – 0.32 | 0.277 |
| Scanner #13 | 0.03 | 0.10 | -0.16 – 0.21 | 0.788 |
| Scanner #14 | 0.02 | 0.09 | -0.15 – 0.19 | 0.839 |
| Scanner #15 | -0.18 | 0.14 | -0.45 – 0.10 | 0.21 |
| Scanner #16 | -0.30 | 0.09 | -0.47 – -0.13 | **0.001** |
| Scanner #17 | 0.26 | 0.11 | 0.05 – 0.48 | **0.016** |
| Scanner #18 | -0.49 | 0.25 | -0.99 – 0.01 | 0.053 |
| Scanner #19 | 0.00 | 0.09 | -0.18 – 0.19 | 0.96 |
| Scanner #20 | -0.83 | 0.92 | -2.63 – 0.97 | 0.365 |
| Scanner #21 | -0.24 | 0.08 | -0.40 – -0.09 | **0.003** |
| Scanner #22 | 0.14 | 0.10 | -0.06 – 0.35 | 0.157 |
| Scanner #23 | 0.19 | 0.10 | -0.01 – 0.39 | 0.063 |
| Scanner #24 | 0.49 | 0.11 | 0.27 – 0.72 | **<0.001** |
| Scanner #25 | -0.05 | 0.08 | -0.20 – 0.11 | 0.553 |
| Scanner #26 | 0.23 | 0.10 | 0.04 – 0.42 | **0.017** |
|  |  |  |  |  |
| Results from the mixed effects Model with BMI as a continuous instead of a categorical variable in the model. |  |  |  |  |
|  |  |  |  |  |
