# Supplementary material for: Examining recent effects of caffeine on default mode network and dorsal attention network anticorrelation in youth
Source: PLoS One. 2025 Jul 2;20(7):e0327385. doi: 10.1371/journal.pone.0327385 (PMC12221008; doi:10.1371/journal.pone.0327385)
Supplement: Supplemental Table 2 — (DOCX) [file pone.0327385.s002.docx]

**Supplemental Document: Supplemental Table 2**

**Parent-Reported Family Income and Adolescent-Reported Caffeine Consumption**

Supplemental bivariable analyses explored parent-reported past 12-month combined family income with the caffeine variables. Chi-Square was conducted to explore the last 24-hour caffeinated beverage consumption with family income. A One-Way Analysis of Variance was conducted to examine weekly caffeinated beverage consumption. The sample size for the supplemental analyses was reduced from 4,673 (in the full paper) to 4,295 because participants refused to answer or were unsure when responding about their past 12-month combined family income. Past 12-month combined family income is a categorical variable with the following values: [a] Less than $5,000, [b] $5,000 through $11,999, [c] $12,000 through $15,999, [d] $16,000 through $24,999, [e] $25,000 through $34,999, [f] $35,000 through $49,999, [g] $50,000 through $74,999, [h] $75,000 through $99,999, [i] $100,000 through $199,999, and [j] $200,000 and more.

**Supplemental Table 2. Income and Caffeinated Beverage in the last 24 hours, N = 4,295**

|  | **Had a Caffeinated Beverage in the last 24 hours, n=683** | | **Did not have a Caffeinated Beverage in the Last 24 Hours, n=3,612** | |
| --- | --- | --- | --- | --- |
| **Income** | **n** | **%** | **n** | **%** |
| Less than $5,000 | 45 | 6.6 | 133 | 3.7 |
| $5,000 through $11,999 | 42 | 6.1 | 119 | 3.3 |
| $12,000 through $15,999 | 31 | 4.5 | 79 | 2.2 |
| $16,000 through $24,999 | 44 | 6.4 | 159 | 4.4 |
| $25,000 through $34,999 | 58 | 8.5 | 207 | 5.7 |
| $35,000 through $49,999 | 84 | 12.3 | 288 | 8.0 |
| $50,000 through $74,999 | 104 | 15.2 | 514 | 14.2 |
| $75,000 through $99,999 | 93 | 13.6 | 488 | 13.5 |
| $100,000 through $199,999 | 140 | 20.5 | 1199 | 33.2 |
| $200,000 and more | 42 | 6.1 | 426 | 11.8 |
| **X2 (p) = 108.1 (<.001); Cramer V (p) = .159 (<.001)** | | | | |
